# The language-ready head: Evolutionary considerations

**DOI:** 10.3758/s13423-016-1087-5

**Published:** 2016-07-01

**Authors:** Cedric Boeckx

**Affiliations:** 1 Catalan Institute for Advanced Studies and Research (ICREA), Barcelona, Spain; 2 Universitat de Barcelona, Gran Via 585, 08007 Barcelona, Spain

**Keywords:** Neurocranium, Language evolution, Genetics, Neurolinguistics

## Abstract

This article offers a succinct overview of the hypothesis that the evolution of cognition could benefit from a close examination of brain changes reflected in the shape of the neurocranium. I provide both neurological and genetic evidence in support of this hypothesis, and conclude that the study of language evolution need not be regarded as a mystery.

Some 40 years ago, Chomsky touched on the topic of language evolution, today still considered by some to be a “mystery,” as opposed to a “problem” (Hauser et al., 2014), and asserted that “[w]e know very little about what happens when 10^10^ neurons are crammed into something the size of a basketball, with further conditions imposed by the specific manner in which the system developed over time” (Chomsky, 1975). Twenty-five years later, Lewontin (1998) notoriously stated that we would never know how cognition evolved.

This essay is motivated by a more optimistic attitude. Obviously, we are still missing many pieces of the puzzle, and figuring out the possible selective factors involved in the evolution of language remains a distant hope. But I find ground for optimism in the search for the underlying developmental and genetic causes of our species’ language faculty, and of its cognitive profile more generally. There are various reasons for this: One is the genomic revolution, which has provided us with a rich set of data not available back in 1975 or 1998. This is critical for setting up constraints on theorizing about the evolution of language. Another reason for optimism is the adoption of a bottom-up approach in comparative psychology, and its appreciation that the basic building blocks of cognition might be shared across a wide range of species, in line with Darwin’s notion of descent-with-modification (De Waal & Ferrari, 2010; Hauser et al., 2002). This bottom-up approach provides the right context for theories that emphasize the generic and elementary character of cognitive operations in the language sciences (Boeckx, 2014; Chomsky, 2005, 2007). A third reason for optimism is the growing body of knowledge linking mind and brain via the mechanism of neural oscillations and the explanatory benefits of their couplings (Buzsaki, 2006; Lisman, 2015). The oscillation-based linking hypothesis is slowly entering the realm of neurolinguistics (Ding, Melloni, Zhang, Tian, & Poeppel, 2016; Friederici & Singer, 2015; Giraud & Poeppel, 2012; Theofanopoulou & Boeckx, in press), but already it holds greater explanatory promise than the classical model inherited from Broca’s and Wernicke’s insights, especially from an evolutionary perspective (Ghazanfar & Poeppel, 2014).

## The hypothesis

Going back to Chomsky’s (1975) statement, it is clear that progress in understanding the evolution of language requires progress in neurolinguistics. Here I feel that linguists have contributed to maintaining language evolution in the realm of mystery by insisting that we know next to nothing about how the brain implements complex cognitive functions like language. Too long an adherence to the classical, Broca-Wernicke model certainly did not help, nor did the field’s insistence on highly domain-specific constructs to characterize linguistic competence. But as we move toward a more adequate neurolinguistics, another aspect of Chomsky’s (1975) statement is worth examining closely. Such an examination will be the focus of this short piece.

I find it significant that Chomsky alluded to both the size and—indirectly, via the basketball metaphor—the shape of the human head, as well as the specific developmental trajectory of “the system.” By *developmental trajectory*, I understand both ontogeny and phylogeny, which in German are jointly referred to as *Entwicklungsgeschichte* (“developmental history”). This happens to be an area where progress has recently been made, as I will show below. In fact, “the specific developmental trajectory of the system” is the context in which I recently put forward a hypothesis concerning language evolution. This hypothesis links the emergence of one of our most distinctive cognitive traits to a well-established anatomical autapomorphy, the globular aspect of our species’ braincase (Boeckx, 2013):

- Globularity hypothesis (*GLBLR*): Globularization Leads to our Brain’s Language-Readiness.

GLBLR departs from more standard speculations pertaining to language evolution, but it does so in ways that I think are beneficial for the prospects of “evolutionary linguistics.” First, it distinguishes between language and language-readiness, stressing the need to understand the anatomical and physiological prerequisites for language acquisition and use (and, of course, the environmental conditions in which these emerged), but also the fact that even when all of this has been characterized, we need to appeal to cultural evolution to capture the complexity of the grammatical systems that are eventually acquired by children. Advocates of cultural evolution readily recognize that any attempt to account for the evolution of linguistic properties through a cultural mechanism must assume the existence of a range of preconditions for the cultural mechanisms to come into play. At least some of these preconditions are to be traced back to neurobiological properties. The term *language-ready brain* is intended to place the focus of investigation on these properties. (This term is to be kept separate from the notion of “protolanguage,” used to refer to an intermediate stage of language evolution, which is arguably appropriate to characterize the cognitive/communicative systems in extinct *Homo* species.)

A second way in which GLBLR departs from more standard approaches to language evolution is that it takes as its target phenotype both brain and braincase (*neurocranium*). This is perhaps the most atypical aspect of the hypothesis. Most accounts of language evolution take as their starting point a behavioral (e.g., “communication”), or cognitive (e.g., “recursion”), or neurological (e.g., “direct cortico-laryngeal connection”) phenotype. GLBLR takes seriously Lieberman’s (2011) arresting statement that we all too often think that apes could evolve to become humanlike without much selection acting on head shape, as our cartoons and movies about talking animals reveal. Yet, in order to think like us, and express themselves like us, other species would have to evolve a brain like ours. Growing such a brain would significantly reshape their craniofacial development and, ultimately lead to a different head shape, making them look like us in relevant respects. GLBLR takes seriously this tightly correlated developmental trajectory between brain and skull, to draw inferences from skull size and shape changes about the organ that generates language. If, as is often said, the mind is what the brain does, the head is how the brain grows. In turn, the head constrains the brain’s growth, nicely illustrating the logic of reciprocal causality that characterizes biology (Lewontin, 2000). According to GLBLR, one cannot characterize the language-ready brain without characterizing the language-ready head.

Taking the head seriously when taking the brain seriously allows us to draw more inferences from the fossil record—as opposed to complaining that soft tissues like the brain do not fossilize, and therefore giving up on bones shedding light on the evolution of language (Hauser et al., 2014). Of course, in order to draw sensible inferences, it is imperative to understand how the correlated growth of the brain and the skull happens, and how this is genetically controlled. But it is not too far-fetched to believe that such an understanding can be achieved. To do so, we can draw on the enormous literature showing how congenital brain and craniofacial defects co-occur. It is now clear that this co-occurrence is a direct consequence of their developmental dependency on common progenitor tissue interactions and signaling pathways during both embryogenesis and early postnatal development. This is why virtually all of the best-known cognitive deficits display dysmorphic features and bone-related problems.

I suspect that one of the main reasons why scientists continue to treat the brain and braincase separately is due to the bad reputation of Gall’s phrenology (even if phrenological tendencies continue to dominate in neuroscience; Uttal, 2001). But I think that Gall was on to something when he tried to deduce mental properties from head shape. What was wrong about his method was the modularity hypothesis guiding his theorizing (Fodor, 1983). An extensive, fully respectable literature on craniofacial growth takes the face as a barometer of the signaling events that modulate brain growth. As DeMyer, Zeman, and Palmer put it in 1964 (p. 256), after studying fetuses with holoprosencephaly, “the face predicts the brain.”

Recent advances in neurolinguistics that appeal to an ever-expanding set of areas for the language network point to a different, but ultimately related, limitation of modular hypotheses about language and the brain (Blank, Balewski, Mahowald, & Fedorenko, 2016; Fedorenko & Thompson-Schill, 2014; Hagoort & Indefrey, 2014). This, it seems to me, is what one should expect if the language faculty provides the scaffolding not only for verbal communication, but also for thought or human-specific cognition (Boeckx, 2011; Chomsky, 2007).

Moving away from modular hypotheses confers to studies on the evolution of language a degree of genericity that allows one to draw on a broad range of animal models for comparative studies, as well as on what we know about other cognitive domains. It also allows one to aim at “low-hanging fruits,” exploiting the extensive literature on gene pathways that support bone growth, suture closings, and so on. All of this contributes to the testability of hypotheses, turning language evolution unambiguously into a problem.

In asserting that “[t]o date, little can be inferred from skull size,” Hauser et al. (2014) were wrong, especially if size is put in connection with shape and developmental trajectory. As Neubauer, Gunz, and Hublin (2010), Gunz et al. (2012), Gunz, Neubauer, Maureille, and Hublin (2010), and Scott, Neubauer, Hublin, and Gunz (2014) have shown, on the basis of data from living primates as well as reconstructed brain development from Neanderthal skulls, the globularization of the braincase is something that sets us apart from out closest extinct and extant relatives. The globularization phase takes place in the first year of life, at a point when the brain is the main driving force behind the shaping of the skull (later on, skull shape is determined by a conjunction of factors that introduce a lot of individual or population variation, and that to some extent obscure the early developmental tight fit between brain and skull; Bruner, Amano, la Cuétara, & Ogihara, 2015).

As Hublin, Neubauer, and Gunz (2015) and Bruner (2010) have shown, this distinct brain growth trajectory reconfigures the brain in several ways. It has been claimed to affect most clearly the parietal lobe, the cerebellum, and the frontal pole; plausibly, as well, it affects the temporal lobe and the olfactory bulbs (Bastir et al., 2011). But our knowledge of brain development, especially the notion of correlated growth, leads us to suspect that other, more deeply embedded structures that leave no direct trace on fossilized skulls also underwent significant changes. Such indirect paleoneurological arguments have been made in recent years concerning the thalamus (Boeckx & Benítez-Burraco, 2014a), the corpus callosum (Theofanopoulou, 2015), or the visual centers in the occipital lobe (Pearce, Stringer, & Dunbar, 2013).

All of these modifications likely contributed to the establishment of a neural circuit capable of supporting the oscillatory regime needed to generate and put to use the complex representations needed for language. For instance, the most conspicuous and uncontroversial anatomical change associated with globularity, which Emiliano Bruner has called “parietal bulging,” quite possibly allowed for the enhancement and transformations of connections rooted in primate cognition. Following a logic that can be traced back to Deacon (1989), it is reasonable to assume that as certain neocortical regions expanded, they allowed for the “invasion” and stabilization of connections that would otherwise have been pruned. Interestingly, there is evidence of significant “parietalization” of certain neural circuits that are highly relevant for language. Thus, Kumar, Croxson, and Simonyan (2016) have argued, on the basis of comparative data, that the evolution of enhanced laryngeal motor-cortex–parietal connections likely allowed for more complex synchrony of higher-order sensorimotor coordination, proprioceptive and tactile feedback, and modulation of learned voice for speech production. In a similar vein, I have argued (Boeckx, 2016) that the expansion of the parietal region significantly enhanced the connection between Broca’s and Wernicke’s regions. There is evidence of a connection between these two regions in nonhuman primates (Bornkessel-Schlesewsky, Schlesewsky, Small, & Rauschecker, 2015; Wilson et al., 2015), but there is also evidence that these two regions connect indirectly through the parietal lobe in humans (Catani & Bambini, 2014; Catani & Jones, 2005; Dick & Tremblay, 2012; Hecht et al., 2013; Mendoza & Merchant, 2014). I have argued that this indirect pairing of Broca’s and Wernicke’s areas via the parietal lobe allowed for the formation of a fronto-parieto-temporal loop that provides the basis for richer representational capacities, namely recursive capacities. It has been independently argued that both fronto-parietal and fronto-temporal networks process sequences. Pairing these two networks could allow for the processing of sequences of sequences, of the sort attested in grammatical constructions. Several authors have independently stressed the relevance (both ontogenetically and phylogenetically) of an indirect, fronto-parieto-temporal pathway for language (de Diego-Balaguer, Martinez-Alvarez, & Pons, 2016; Friederici, 2012). I claim that this fronto-parietal “dorsalization” of the language network is something that globularization could have boosted.

This enhanced language network could recruit specific parts of subcortical structures (e.g., thalamus and cerebellum) that were also affected by globularization. This naturally leads to the expectation that some of the most distinct aspects of language processing are distributed over an extended cortico-subcortical language network, which appears to be the case, for instance, for syntax (Blank et al., 2016).

Another aspect of GLBLR worth mentioning is the exploration of the crosstalk between neurogenesis and osteogenesis, which has already yielded several genes of interest (Benítez-Burraco & Boeckx, 2015; Boeckx & Benítez-Burraco, 2014a, 2014b, 2015), many of which show signs of positive selection in anatomically modern humans, such as *RUNX2*. A sizeable literature now shows that genes critical to craniofacial development, such as *SATB2* or *DYRK1A*, give rise to severe intellectual deficits and impaired speech when mutated (Ji et al., 2015; Liedén, Kvarnung, Nilssson, Sahlin, & Lundberg, 2014). This literature suggests that we would do well to examine closely the “bone-related” genes found in human-accelerated regions of the genome (Gittelman et al., 2015), or those exhibiting fixed mutations in anatomically modern humans relative to Neanderthals/Denisovans (Pääbo, 2014), and try to determine whether these may have played an important role in shaping our brain. Likewise, it would be very productive to study the role played by robust “language-related” genes (Lai et al., 2001 *et seq.*) in craniofacial development. Some recent studies appear very promising, in this respect. Thus, it has recently been shown that *FOXP2* is implicated in the development of the jaw (Cesario, Almaidhan, & Jeong, 2016). Direct interactions between *FOXP2* and *RUNX2* have been demonstrated (Zhao et al., 2015). It is also worth noting that some of *FOXP2*’s most direct targets affect bone growth. For instance, this is the case with *ROR2* (see Vernes et al., 2007, and Konopka et al., 2009, on this gene’s connection with *FOXP2*). When mutated, *ROR2* gives rise to Robinow syndrome, from which both physical and cognitive anomalies arise. Likewise, *FOXP1*, another robust “language-related” gene, is implicated in a syndrome (*FOXP1 deficiency syndrome*) that not only involves language problems, but also dysmorphic features (Lozano, Vino, Lozano, Fisher, & Deriziotis, 2015). The same holds for *CNTNAP2* (Rodenas-Cuadrado et al., 2016), another close partner of *FOXP2*.

## Conclusion

All in all, I feel that taking advantage of the coevolution of brain and braincase, as GLBLR does, offers a fruitful way to address the question of how the human brain organized itself to generate modern cognition. True, we may not know everything about “what happens when 10^10^ neurons are crammed into something the size of a basketball, with further conditions imposed by the specific manner in which the system developed over time,” but we already know enough to claim that growing a primate brain that looks like a basketball likely mattered (and matters) a lot. In addition to drawing on the literature on mental and speech disorders, we can, as Weidenreich (1941) suggested long ago, exploit animal studies that have shown brain reorganization associated with skull reorganization (see Aristide et al., 2016, for monkeys; Carril, Tambussi, Degrange, Benitez Saldivar, & Picasso, 2015, for parrots; and McGreevy et al., 2013; Roberts, McGreevy, & Valenzuela, 2010; and Stone, McGreevy, Starling, & Forkman, 2016, for dogs). We can also link GLBLR to other hypotheses linking skull changes and brain/cognition/behavior changes, such as those formulated in the context of the domestication syndrome (Benítez-Burraco, Theofanopoulou, & Boeckx, in press; Wilkins, Wrangham, & Fitch, 2014). All of these connections will enable us to draw on a broader comparative basis to better articulate our hypotheses and to make well-defined, testable predictions—a sure sign that we are dealing with a problem, not a mystery.
